# A life-threatening case of pseudo-aldosteronism secondary to excessive liquorice ingestion

**DOI:** 10.1186/s12902-021-00816-4

**Published:** 2021-08-06

**Authors:** Joseph McHugh, Ramesh Nagabathula, Ma Pyeh Kyithar

**Affiliations:** 1 Endocrinology Department, Midlands Regional Hospital Portlaoise, Co. Laois, Republic of Ireland; 2 Radiology Department, Midlands Regional Hospital Portlaoise, Co. Laois, Republic of Ireland

**Keywords:** Liquorice, Glycyrrhizin, 11-beta-hydroxysteroid dehydrogenase, Hypokalaemia, Hyperaldosteronism, Pseudo-aldosteronism, Hypertension, Case report

## Abstract

**Background:**

Liquorice is found in many food products, soft drinks, and herbal medicines. Liquorice ingestion is an uncommon cause of apparent mineralocorticoid excess or pseudo-aldosteronism. The mechanism involves the inhibition of 11-beta-hydroxysteroid dehydrogenase type-2 by the active ingredient called glycyrrhizin. This leads to the uninhibited activation of mineralocorticoid receptors by cortisol. Confectionary products that contain liquorice are readily available in many countries around the world.

**Case presentation:**

We report a case of severe refractory hypokalaemia with hypertensive crisis and acute pulmonary oedema due to excessive liquorice consumption. A 79-year-old female presented to the emergency department following a road traffic accident. She described feeling weak and dizzy while driving before the collision. She attended her general practitioner (GP) several weeks earlier for fatigue and was being managed for hypokalaemia on oral potassium supplements. Investigations revealed hypertension (BP 180/69 mmHg), severe hypokalaemia (K 2.2 mmol/l), normal renal function, normal serum magnesium with metabolic alkalosis. Spot urinary potassium was 22 mmol/l. The patient denied taking medications including over-the-counter or herbal medication that can cause hypokalaemia. Hypokalaemia persisted despite aggressive intravenous (i.v.) and oral potassium replacement. She later developed a hypertensive crisis (BP 239/114 mmHg) with pulmonary oedema. She required admission to the intensive care unit and was managed with intravenous furosemide infusion and isosorbide dinitrate infusion. On further discussion, our patient admitted to struggling with nicotine cravings since quitting smoking two months earlier. She began eating an excessive amount of liquorice sweets to manage her cravings. Suppression of plasma renin and aldosterone supported the diagnosis of apparent mineralocorticoid excess secondary to excessive liquorice consumption. Her symptoms and hypokalaemia resolved after stopping liquorice intake.

**Conclusions:**

This case highlights the life-threatening and refractory nature of hypokalaemia secondary to excessive liquorice consumption. This case also emphasizes the importance of comprehensive history taking including dietary habits. Increased awareness among the public is required regarding the potential health hazards of excessive liquorice consumption.

## Background

Liquorice-induced hyperaldosteronism has been well described in multiple previous case reports. These involve sweets, gum, and beverages containing liquorice root extract. The clinical history commonly describes muscle weakness, periodic paralysis, severe hypertension, headaches, and hypokalaemia. Occasional cases describe severe and life-threatening effects of liquorice ingestion including; tetra-paresis [1], hypertensive encephalopathy [2], and pulmonary oedema [3]. A recent report in the New England Journal of Medicine described a case of pseudo-aldosteronism complicated by cardiac arrest associated with ventricular fibrillation due to metabolic, renal, vascular, and cardiotoxic effects from apparent mineralocorticoid excess due to liquorice consumption [4].

## Case presentation

A 79-year-old female presented to the emergency department following a road traffic accident. She described feeling weak while driving before the collision. She was wearing a seatbelt and was uninjured.

Her medical history included chronic obstructive pulmonary disease, dyslipidaemia, constipation, hypertension and she was an ex-smoker as she quit smoking two months earlier. Her medications included Lercanidipine 10 mg, Aspirin 75 mg, Ezetimibe/Simvastatin 10 mg/20 mg, Lactulose as required, and Indacaterol/Glycopyrronium 85mcg/43mcg.

She had attended her GP on multiple occasions over the previous two months feeling generally weak and lethargic. She was noted to be hypertensive, requiring increasing doses of her antihypertensive medications. Laboratory tests with her GP showed hypokalaemia and she was commenced on oral potassium supplements.

**Fig. 1:**
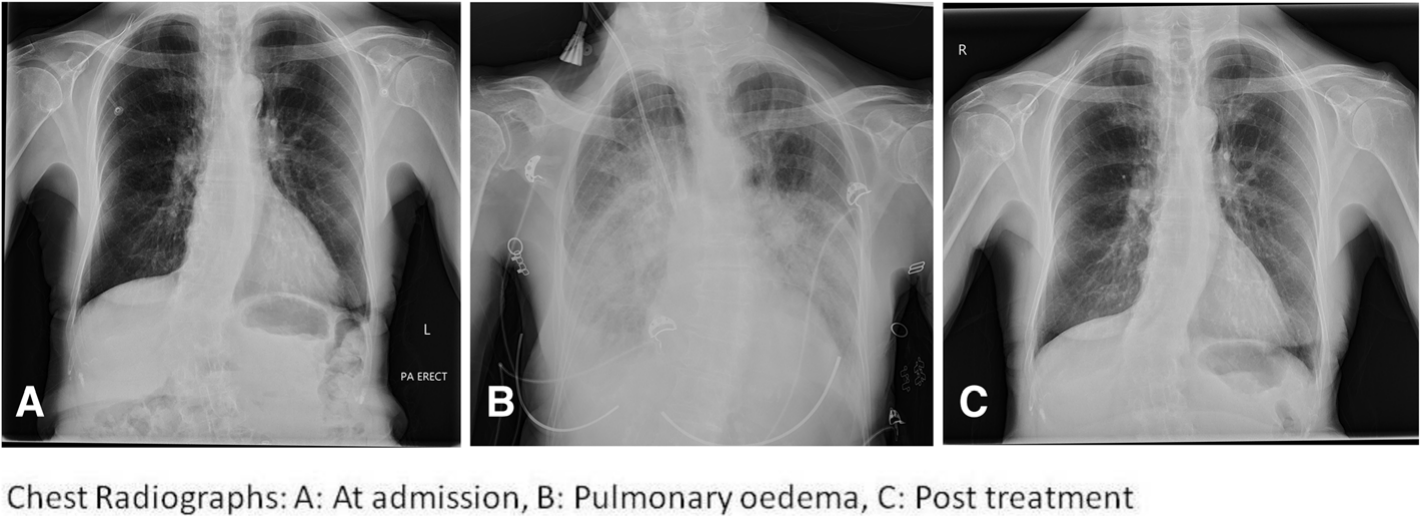
Chest Radiographs: **A** At admission, **B** Pulmonary oedema, **C** Post treatment

On our initial assessment she was hypertensive, blood pressure 180/69. She was of slim build (body mass index(BMI) of 20.2 kg/m^2^) with no cushingoid features, clinical examination was unremarkable and there was no traumatic injury. ECG at presentation showed sinus rhythm with left axis deviation, left bundle-branch block, and QT prolongation of 502 ms. Labs revealed severe hypokalaemia (K 2.2 mmol/l), normal renal function (Urea 3.4 mmol/L, Creatinine 54ummol/L, Na 143 mmol/L), normal serum magnesium (Mg 0.79 mmol/L) with metabolic alkalosis (pH 7.53, PCO2 5.4 (40.3mmhg), PO2 7.1 (53.3mmhg), HCO3 34). Spot urinary potassium was 22 mmol/l. Chest x-ray on admission showed mild cardiomegaly, and there was no active lung disease (Fig. 1). CT Brain reported no acute findings.

Our impression was severe hypokalaemia and hypertension with metabolic alkalosis secondary to hyperaldosteronism. Differential diagnoses included primary hyperaldosteronism, secondary hyperaldosteronism, or due to medications/food. Further workup was sent including; plasma renin, serum aldosterone, urinary electrolytes. She was managed with aggressive potassium replacement, requiring 80 mmol i.v. potassium chloride (KCl) daily for four days (total 240 mmol i.v. KCl) to achieve a serum potassium level above 3.5 mmol/L. Her systolic blood pressure settled below 150mmhg.

**Fig. 2:**
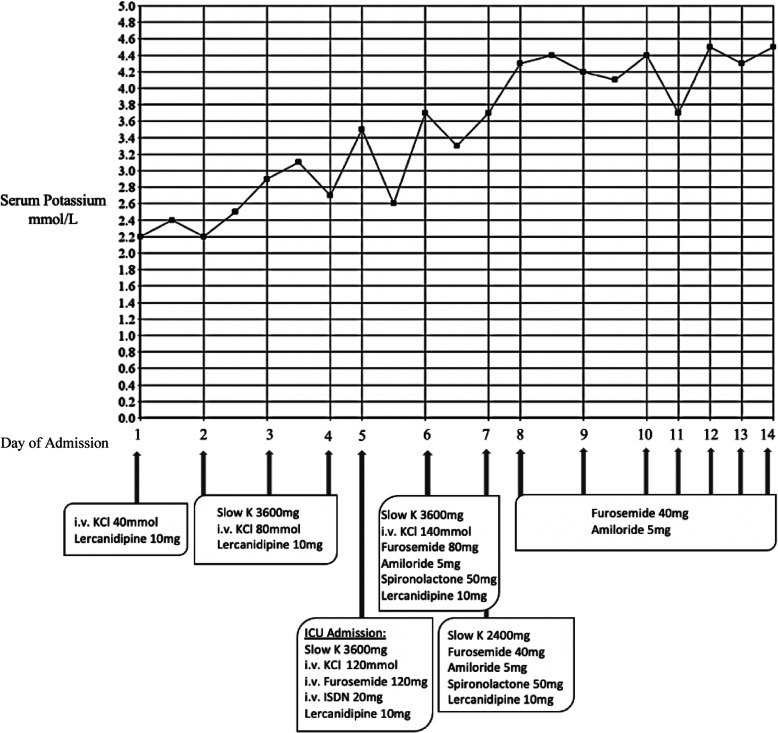
Demonstrates the serum potassium each day during admission with the dose of i.v. and oral medications used

On day five of her admission, our patient developed a hypertensive emergency (BP 239/114 mmHg) with acute pulmonary oedema requiring admission to the intensive care unit (ICU). Chest X-ray demonstrated bilateral air space opacifications and perihilar haze, with bilateral pleural effusions in keeping with pulmonary oedema (Fig. 1). She was managed with i.v. Furosemide 160 mg/24 h, Isosorbide dinitrate infusion 2.5 mg/hr for 8 h, and oral spironolactone 50 mg once daily. Pulmonary oedema resolved within 24 h and blood pressure improved to 130/55 mmHg. She was changed from i.v. furosemide to oral Furosemide/Amiloride 40/5 mg. Echocardiogram showed normal left ventricle size and function, paradoxical septal motion consistent with left bundle branch block, mildly dilated left atrium, and right ventricular systolic pressure less than 35 mmHg. On review of her imaging, a recent CT thorax was noted to include the adrenal glands. This was reviewed and reported no evidence of adrenal adenomas or hyperplasia Fig. 2.

We revisited the clinical history. On further questioning, she reported struggling with nicotine cravings since quitting smoking two months earlier. She began eating excessive amounts of liquorice sweets to satisfy her nicotine cravings. The liquorice sweets she was eating contained 4% liquorice root extract. The estimated intake was 1.14gm-2.28gm liquorice extract per day for the previous two months. She continued to eat liquorice while in hospital until admission to the ICU. She was advised to stop consuming liquorice once the medical team became aware.

Further investigations returned. Her urinary potassium was noted to be inappropriately normal [24 h Urinary Potassium: 57.3 (25–125) mmol/24 h] in the setting of severe hypokalaemia with high urinary sodium [24 h Urinary Sodium: 353.4 pg/ml(40-220 pg/ml)]. Elevated urinary sodium excretion was likely due to diuretic therapy and the production of natriuretic peptides in response to hypervolemia. Plasma renin and serum aldosterone taken day three of admission were both suppressed [Plasma renin 4.4 pg/ml(< 20 pg/ml), Serum Aldosterone < 26 pg/ml (42–209 pg/ml)] suggestive of an extra-adrenal mimic causing a pseudo-aldosteronism. This was in keeping with a diagnosis of acquired apparent mineralocorticoid excess secondary to excessive Liquorice ingestion. Her serum potassium and systolic blood pressure began to normalise following the cessation of Liquorice. Furosemide/Amiloride was reduced to 20 mg/5 mg during admission and was discontinued 10 days after discharge. Her serum potassium remained within the normal range in the following weeks and her blood pressure normalised. Her follow-up ECG demonstrated sinus rhythm with left axis deviation and left bundle-branch block, and QTc improved to 458 ms.

## Discussion and conclusion

The mineralocorticoid receptor is present within various tissues throughout the body. In the kidneys, it acts in the distal convoluted tubule. Activation of the mineralocorticoid receptors leads to the expression of epithelial sodium channels, regulating intravascular volume via retention of sodium and water with the excretion of potassium. In vitro, mineralocorticoid receptors will bind cortisol and aldosterone with equal affinity [5]. Cortisol is present in the serum at substantially higher concentrations. 11 beta-hydroxysteroid dehydrogenase type 2 (11βHSD2) is an enzyme expressed by aldosterone-specific tissue and leads to the conversion of active cortisol to inactive cortisone. This prevents the inappropriate activation of mineralocorticoid receptors by cortisol, conferring ligand specificity to the mineralocorticoid receptor [6].

Liquorice contains the compound glycyrrhetinic acid which inhibits 11βHSD2 by competitive inhibition and reduction in gene expression. This allows the unopposed activation of mineralocorticoid receptors by cortisol, leading to an apparent mineralocorticoid excess in the setting of a normal or suppressed serum aldosterone [7].

Pulmonary oedema as a consequence of liquorice ingestion is rarely described. A PUBMED search of the terms “Liquorice” + “Pulmonary oedema” revealed two previously reported cases of pulmonary oedema associated with liquorice ingestion. The first case described a 64-year-old male in 1997 who presented with acute pulmonary oedema following a liquorice binge (3.6gm glycyrrhetinic acid). He was treated acutely with diuresis and recovered within two days. He was found to have no cardiac abnormality and recovered without relapse following avoidance of liquorice [3]. A second case, reported in 2010, involved a 66-year-old male ex-alcoholic who was managing his alcohol cravings with liquorice beverages(1.6gm glycyrrhetinic acid daily). He presented with typical findings of muscle weakness, hypokalaemia, metabolic alkalosis, clinical exam on admission noted euvolemia. Similar to our case, he was treated with aggressive i.v. saline 0.9% containing K + 54 mmol/L. Twenty hours into his admission he developed acute pulmonary oedema requiring ICU admission and management with i.v. diuresis and nitrates. He made a good recovery and remained well following abstinence from liquorice containing products [8]. These cases mirror the clinical course of our patient where liquorice appears to be the primary precipitating factor for acute life-threatening pulmonary oedema (Table 1).

**Table 1:** Comparison of our case with previously reported cases of pulmonary oedema as a consequence of excess liquorice ingestion

	Our case:	Caubet-Kamar, Natacha, et al. 2010:	Chamberlain, James J 1997:
Demographics	79 year-old female	66 year-old male	64 year-old male
Pre-morbid status	COPD HTN Constipation Ex-smoker	Chronic alcohol excess Smoker	“Previously healthy”
Liquorice ingestion	1.7gm/day × 2 months	1.6gm/day × 2 months	3.6gm over 3 days
Lowest serum potassium	2.2 mmol/l	1.8 mmol/l	3.5 mmol/l
Peak blood pressure	239/114 mmHg	200/100 mmHg	180/80 mmHg
Treatment given	i.v KCl PO KCl PO Lercanidipine PO Furosemide/amiroride PO Spironolactone i.v. Furosemide i.v. ISDN	i.v. KCl PO Spironolactone i.v. Furosemide i.v. ISDN	i.v Furosemide PO Enalapril PO KCl
Outcome	Treatment stopped 10-days after discharge Remained normotensive and normal serum potassium at 8 weeks	Continued furosemide and spironolactone on discharge Normotensive and normal serum potassium at 1 month. Changed to PO Amlodipine 5 mg alone	Treatment stopped before discharge Normal serum potassium and normotensive at 2 months

It remains unclear which factors determine a patient’s response to liquorice ingestion or who may be more sensitive to its effects. Liquorice appears to have many different effects of varying severity in different people. Penninkilampi et al. 2017 published a systemic review and meta-analysis which aimed to correlate the degree of liquorice ingestion with hypokalaemia and hypertension. This involved 18 studies (*n* = 337). They found a significant correlation between the level of liquorice ingestion with an elevation of systolic and diastolic blood pressure. Hypokalaemia and suppressed renin and aldosterone were also associated with liquorice ingestion, however, this did not appear to be in a dose-dependent manner [9]. René K. Støving et al. 2011 suggest that anorexia nervosa confers increased sensitivity to glycyrrhetinic acid, however, this is based on a single anecdote and may not be of significance [10]. A 2001 study aimed to investigate the dose–response relationship, the time-response relationship, and the inter-individual response variability to glycyrrhetinic acid. They found a dose-dependent rise in systolic blood pressure with as little as 50gm Liquorice per day (75 mg glycyrrhetinic acid). They reported no inter-individual response variability with the individual response following a normal distribution [11]. This study was limited by its homogenous patient population, it included only healthy adults with an age range of 20-43 year-old, no data was given regarding participant ethnicity. In our case, her age and low normal BMI might be the predisposing factors for the effects of glycyrrhetinic acid from liquorice [10]. We also felt that her ongoing liquorice ingestion, together with the large volume of intravenous fluids required to replace her ongoing potassium loss, precipitated the hypertensive crisis.

This case highlights the life-threatening and refractory nature of hypokalaemia secondary to excessive liquorice consumption. It stresses the importance of comprehensive history taking, including dietary intake, in assessing patients with hypokalaemia. Increased awareness among the public is required regarding the potential health hazards of excessive liquorice consumption.

## Data Availability

All data generated or analysed during this study are included in this published article.

## Abbreviations

HTN: Hypertension
BP: Blood Pressure
Na: Sodium
K: Potassium
GP: General Practitioner
i.v.: Intravenous
BMI: Body mass index
